# Uncontrolled Primary Hypothyroidism Precipitating Non-cirrhotic Hyperammonemia: A Unique Interplay Between the Liver and Thyroid

**DOI:** 10.7759/cureus.57861

**Published:** 2024-04-08

**Authors:** Raghav Bassi, Sushil Rayamajhi, Kobina Essilfie-Quaye, Jasmay Randhawa, Sripal Padam

**Affiliations:** 1 Internal Medicine, University of Central Florida College of Medicine, Graduate Medical Education, Hospital Corporation of America (HCA) Florida North Florida Hospital, Gainesville, USA; 2 Internal Medicine, University of Central Florida College of Medicine, Graduate Medical Education, Hospital Corporation of America (HCA) Florida West Florida Hospital, Pensacola, USA; 3 Internal Medicine, University of Central Florida College of Medicine, Gainesville, USA

**Keywords:** altered mental status evaluation, nonalcoholic fatty liver disease (nafld), non-cirrhotic hyperammonemia, overt hypothyroidism, hepatic encephalopathy (he)

## Abstract

Hepatic encephalopathy is typically seen in advanced liver disease and in patients with a transjugular intrahepatic portosystemic shunt. Common triggers include infections, gastrointestinal bleeding, electrolyte disturbances, dehydration, and drug/toxin use such as benzodiazepines and alcohol. In rare instances, other metabolic abnormalities such as hypothyroidism may also exacerbate hyperammonemia in patients with underlying liver disease due to hypothyroidism-induced myopathy, which increases urea production and decreases clearance through reduced glutamine synthetase activity. We present the case of a 60-year-old female who presented with markedly elevated thyroid stimulating hormone, reduced free thyroxine, and elevated serum ammonia levels. Although lactulose and rifaximin were initially started, her symptoms did not clinically improve until the underlying cause of her hyperammonemia was treated. Levothyroxine was initiated, and she reported rapid clinical improvement in her symptoms. Hyperammonemia carries a 40% mortality rate, and therefore clinicians need to be aware of this rare but intricate relationship between advanced liver disease and hypothyroidism for the prompt diagnosis and management of this condition.

## Introduction

Hepatic encephalopathy (HE) is seen in advanced liver disease and occurs in roughly 45% of patients with cirrhosis and 24-53% of patients with a transjugular intrahepatic portosystemic shunt [1]. Common triggers include infections, gastrointestinal bleeding, electrolyte disturbances, dehydration, and drug/toxin use such as benzodiazepines and alcohol. The underlying pathophysiology is characterized by the accumulation of neurotoxic substances such as ammonia in the bloodstream that ultimately cross the blood-brain barrier, resulting in personality changes, a decreased level of consciousness, and confusion. In severe cases, HE may lead to coma and ultimately death [1,2]. The liver plays an important role in the removal of ammonia; however, in the setting of decreased functioning hepatocytes, as seen in cirrhosis or portosystemic shunting, hyperammonemia can occur. In rare instances, non-cirrhotic hyperammonemia can also precipitate altered mental status from the accumulation of ammonia in the blood in the setting of normal liver function. Metabolic abnormalities, such as hypothyroidism, may exacerbate non-cirrhotic hyperammonemia in patients with normal liver function, with only a couple of cases reported in the literature. In addition, hypothyroidism shares similar symptoms to overt hyperammonemia, such as memory impairment, mental slowing, weakness, myalgias, edema, and ascites, and should remain a differential diagnosis in patients presenting with altered mental status [2,3]. We present an interesting case of a patient who developed non-cirrhotic hyperammonemia from uncontrolled hypothyroidism and hopes to contribute to our current understanding of this rare but important disease process as prompt recognition and early therapeutic intervention yield good clinical outcomes.

## Case presentation

A 60-year-old Caucasian female with a past medical history of type 2 diabetes mellitus, class III obesity, and primary hypothyroidism presented to the emergency department for a chief complaint of acute altered mental status that started one day prior to admission. On presentation, she was afebrile and hemodynamically stable. Examination revealed a morbidly obese female that only moaned to sternal rub and moved all extremities spontaneously. Physical examination did not reveal any evidence of jaundice, splenomegaly, hepatomegaly, palmar erythema, nuchal rigidity, or ascites. After speaking with the patient's family, there was no obvious evidence of alcohol use, melena, hematochezia, coffee-ground emesis, or the recent use of chemotherapy agents. Labs revealed a normal, complete blood count. The comprehensive metabolic panel was remarkable for an elevated blood urea nitrogen of 27 mg/dL, elevated aspartate aminotransferase of 175 U/l, and alanine aminotransferase of 72 U/l. The serum ammonia concentration was also significantly elevated at 230 μmol/L, as seen in Table 1. A non-contrast CT of her head was obtained, which did not reveal any hemorrhage, mass effect, or cerebral edema. Ultrasound of the abdomen revealed a normal liver and spleen with no evidence of portal hypertension. Empiric treatment with rectal lactulose and rifaximin was initiated and titrated to three to four bowel movements a day, with minimal improvement in her altered mental status. Given her hyperammonemia on presentation and lack of clinical improvement, urine toxicology, full viral hepatitis panel, antinuclear antibody, anti-actin antibody, alpha-fetoprotein, and ceruloplasmin were all ordered to evaluate for secondary underlying metabolic etiologies of her acute encephalopathy. All of her labs were unremarkable. Vitamin B12, serum thiamine, rapid plasma reagin, thyroid-stimulating hormone (TSH), and free thyroxine (T4) were also ordered to examine other possible etiologies of her altered mental status, such as hypothyroidism, Wernicke encephalopathy, and syphilis. All her labs came back unremarkable except for a significantly elevated TSH at 171 mU/l and a low-free T4 at 0.14 ng/dL, as seen in Table 1. Upon further questioning, it was determined that she had stopped taking her levothyroxine a month prior to admission due to a change in her insurance. She was restarted on levothyroxine at 200 mcg with a rapid improvement in her mental status within days. Her TSH and ammonia were both rechecked 48 hours after the initiation of levothyroxine, and they both significantly improved. She was then discharged home in stable and improving condition, with close follow-up with her primary care physician.

**Table 1:** Labs were both on admission and 48 hours after receiving levothyroxine MCV: mean corpuscular volume; BUN: blood urea nitrogen; GFR: glomerular filtration rate; AST: aspartate aminotransferase; ALT: alanine aminotransferase; TSH: thyroid-stimulating hormone; T4: thyroxine

Tests	Results on admission	Results after levothyroxine	References	Units
White blood count	4.8	4.9	4.0-10.5	10³ uL
Hemoglobin	12.3	12.3	13.7-17.5	g/dL
Hematocrit	36.4	37.2	40.1-51	%
Platelets	62	55	150-400	10³ uL
MCV	93.1	94.7	79.0-92.2	fl
Sodium	137	139	136-145	mmol/L
Potassium	5.1	4	3.5-5.1	mmol/L
Chloride	106	110	98-107	mmol/L
Carbon dioxide	25	26	21-32	meq/L
Glucose	159	95	74-106	mg/dL
BUN	27	13.1	7-18	mg/dL
Creatinine	2.01	1.53	0.6-1.30	mg/dL
GFR	30	34	0-120	mL/min
Albumin	2.5	2.6	3.4-5.0	g/dL
Calcium	8.8	8.7	8.5-10.1	mg/dL
Phosphorus	4.7	3.8	2.5-4.9	mg/dL
Total bilirubin	0.5	1.1	0.2-1.0	mg/dL
AST	175	148	15-37	units/L
ALT	72	68	13-56	units/L
Magnesium	1.6	1.7	1.5-2.4	mg/dL
Lactic acid	0.58	N/A	0.4-2.0	mmol/L
TSH	171	50	0.358-3.740	uIU/mL
Free T4	0.14	0.33	0.76-1.46	ng/dL
Serum ammonia	230	138	11-32	umol/L

## Discussion

Hypothyroidism exhibits symptoms similar to those of HE and should be included in the differential diagnoses for altered mental status. However, there is limited data on whether hypothyroidism can both occur and trigger hyperammonemia. This unique case illustrates the intricate interplay between the liver and thyroid functions and supports the role of hypothyroidism as a precipitating factor in triggering non-cirrhotic hyperammonemia through increased urea production and decreased clearance. The presence of elevated ammonia levels, especially without other signs of liver decompensation, should raise suspicion for hypothyroidism in such cases. Other differential diagnoses that must also be considered by clinicians include intracranial lesions (subdural hematomas, tumors, strokes, and abscesses), central nervous system infections (meningitis, syphilis), metabolic encephalopathies (hypoglycemia, infection), Wernicke encephalopathy, post-seizure encephalopathy, and drug-related encephalopathy, which were all ruled out in the case above.

When a patient presents with hyperammonemia, it is essential to determine if it is primary or secondary in origin. Primary hyperammonemia occurs from congenital enzyme deficiencies in the urea cycle, including ornithine transcarbamoylase and argininosuccinate lyase [3]. Secondary hyperammonemia can then be broken down into either hepatic or non-hepatic [3]. Non-cirrhotic etiologies of hyperammonemia include disruptions in the mitochondrial pathway from drug toxicity like cyanide, carbamazepine, valproic acid, and iron. Furthermore, common chemotherapeutic agents like cytarabine, vincristine, amsacrine, etoposide, L-asparagine, cyclophosphamide, and 5-fluorouracil have also been implicated in non-cirrhotic hyperammonemia [3]. Our patient above did not report taking any chemotherapy medications and denied any new medications.

In addition to medications, other causes of non-cirrhotic hyperammonemia include infections from urease-producing organisms such as *Proteus mirabilis*, *Klebsiella*, and *Escherichia coli*, especially in the setting of partial urea cycle enzyme deficiencies, and surgeries such as gastric bypass [3]. There have also been reported cases of hypothyroidism precipitating hyperammonemia and negatively impacting liver structure and function. Although poorly understood, it is postulated that this occurs through hypothyroidism-induced myopathy, which increases urea production from muscle breakdown and decreases clearance through reduced glutamine synthetase activity from hepatocellular damage [1,2].

Distinguishing encephalopathy from myxedema coma from hypothyroidism versus non-cirrhotic hyperammonemia can be challenging, especially in patients with concurrent liver diseases, as symptoms of both conditions overlap. Fatigue, mental status changes, weakness, myalgias, and shortness of breath can be present in both disorders. Swelling, fluid buildup in the abdomen (ascites), and pleural effusion can also occur in both conditions [3,4]. Although they both have similar clinical presentations, severe hyperammonemia would not be expected in myxedema coma. Furthermore, our patient did not have any evidence of thickening or non-pitting edema, which is typically observed in myxedema coma.

The diagnosis of non-hepatic hyperammonemia is a diagnosis of exclusion and requires a high index of suspicion [3]. In this particular case, although severe hypothyroidism might have also contributed to the coma, it is unlikely to have been the sole cause of her encephalopathy. Profound hyperammonemia was observed upon admission without other signs of liver dysfunction, such as abnormal blood clotting, jaundice, or fluid accumulation in the abdomen. The impact of T4 on ammonia metabolism is not well understood. Although protein synthesis decreases in hypothyroidism, urea production, and urea cycle enzyme activities increase in experimental hypothyroidism in rats [4,5,6]. These findings suggest that hypothyroidism might enhance ammonia production. Another reported case involving patients who developed HE from cirrhosis showed slowing and triphasic waves on electroencephalograms, which were similarly observed in patients with hypothyroid-induced hyperammonemia, indicating the intricate relationship between these two disease processes [7]. This pattern is rare in overt hypothyroidism, which typically demonstrates diffuse slowing without specific waves [8]. Furthermore, current guidelines from the American Association for the Study of Liver Disease do not recommend routinely checking ammonia levels in patients with encephalopathy; however, a normal ammonia value in patients with encephalopathy can be of diagnostic value, making HE unlikely [9]. Recent studies have also revealed no correlation between the severity of altered mental status and ammonia levels; however, underlying concomitant conditions like hypothyroidism may complicate this intricate relationship, as seen in the case above. Furthermore, our patient's serum ammonia levels and clinical symptoms didn’t improve until levothyroxine was restarted, suggesting that the underlying etiology of her hyperammonemia was likely from underlying uncontrolled hypothyroidism.

Treatment for non-cirrhotic hyperammonemia includes the proper identification and treatment of the underlying cause. Antibiotics are frequently given as infections are common precipitating factors. These patients should also be followed up closely on discharge, as HE carries a 40% mortality rate [8]. Mental status changes must be monitored carefully with a multidisciplinary team approach, including dieticians for adequate calorie monitoring, home care nurses, and physical therapists [8,9]. Given that cirrhosis causes 90% of hyperammonemia, treating patients with cirrhosis who develop hyperammonemia with lactulose and rifaximin is warranted [10]. If patients are refractory to medical therapy, then evaluating and treating underlying metabolic and infectious processes should be done, as seen in our case above.

## Conclusions

Distinguishing acute encephalopathy from underlying acute hypothyroidism or non-cirrhotic hyperammonemia in the setting of normal liver function can be challenging. In patients with normal liver function, elevated ammonia levels in the setting of an elevated TSH and low free T4 with changes in acute mental state as seen in the case above can be attributable to hypothyroidism-induced non-cirrhotic hyperammonemia. Diagnosis is challenging and is a diagnosis of exclusion after ruling out more common causes of encephalopathy. Treatment is aimed at prompt identification of the underlying cause and has a good prognosis if treated immediately.

## References

[1] Rudler M, Weiss N, Bouzbib C, Thabut D (2021). Diagnosis and management of hepatic encephalopathy. Clin Liver Dis.

[2] L'age M, Meinhold H, Wenzel KW, Schleusener H (1980). Relations between serum levels of TSH, TBG, T4, T3, rT3 and various histologically classified chronic liver diseases. J Endocrinol Invest.

[3] Odigwe CC, Khatiwada B, Holbrook C, Ekeh IS, Uzoka C, Ikwu I, Upadhyay B (2015). Noncirrhotic hyperammonemia causing relapsing altered mental status. Proc (Bayl Univ Med Cent).

[4] Huang MJ, Wu SS, Liaw YF (1994). Thyroid abnormalities in patients with chronic viral hepatitis. Hepatology.

[5] Marti J, Portoles M, Jimenez-Nacher I, Cabo J, Jorda A (1988). Effect of thyroid hormones on urea biosynthesis and related processes in rat liver. Endocrinology.

[6] Cheema-Dhadli S, Jungas RL, Halperin ML (1987). Regulation of urea synthesis by acid-base balance in vivo: role of NH3 concentration. Am J Physiol.

[7] Hitoshi S, Terao Y, Sakuta M (1993). Portal-systemic encephalopathy and hypothalamic hypothyroidism: effect of thyroid hormone on ammonia metabolism. Intern Med.

[8] Markand ON (1984). Electroencephalography in diffuse encephalopathies. J Clin Neurophysiol.

[9] Deutsch-Link S, Moon AM, Jiang Y, Barritt AS 4th, Tapper EB (2022). Serum ammonia in cirrhosis: clinical impact of hyperammonemia, utility of testing, and national testing trends. Clin Ther.

[10] Kalra A, Norvell JP (2020). Cause for confusion: noncirrhotic hyperammonemic encephalopathy. Clin Liver Dis (Hoboken).

